# Supplementary material for: Prevalence and Characteristics of Phenicol-Oxazolidinone Resistance Genes in Enterococcus Faecalis and Enterococcus Faecium Isolated from Food-Producing Animals and Meat in Korea
Source: Int J Mol Sci. 2021 Oct 20;22(21):11335. doi: 10.3390/ijms222111335 (PMC8583520; doi:10.3390/ijms222111335)
Supplement: Supplementary file 1 [file ijms-22-11335-s001.zip › ijms-1415638-supplementary.pdf]

Table S1. Location of antimicrobial resistance genes identified in the three *E. faecalis* genomes

Strain	Genetic context	ARGs	Identity (%)	Position (bp)	Predicted phenotype	Accession no.
EFS17	Chromosome	*lsaA*	98.7	569906..571402	lincomycin	AY737526
		optrA	100.0	745279..747246	linezolid	KT862783
		fexA	99.6	747934..749361	chloramphenicol	AJ549214
		ermA	100.0	751577..752170	erythromycin	EU348758
		ant(9)-Ia	100.0	752340..753122	spectinomycin	X02588
	Plasmid	*ermB*	100.0	10120..10857	erythromycin	X72021
		aph(3')-III	100.0	11607..12401	kanamycin	M26832
		ant(6)-Ia	100.0	13033..13941	streptomycin	AF330699
		lnuB	100.0	17449..18252	lincomycin	JQ861959
		lsaE	100.0	18306..19790	lincomycin	JX560992
		ant(6)-Ia	99.9	23369..24127	streptomycin	KF421157
		aac(6')-aph(2'')	100.0	24291..25730	gentamicin	M13771
		ermB	100.0	26557..27294	erythromycin	U18931
		dfrG	100.0	29363..29848	trimethoprim	AB205645
		ermB	100.0	33793..34530	erythromycin	X72021
		cat	98.9	42659..43306	chloramphenicol	U35036
		tetL	100.0	44348..45724	tetracycline	M29725
		tetM	96.5	45918..47837	tetracycline	EU182585
		str	99.8	74376..75224	streptomycin	FN435330
EFS36	Chromosome	*lsaA*	99.4	1932120..1933616	lincomycin	AY737526
	Plasmid (pEF36_1)	*aac(6')-aph(2'')*	99.9	3310..4749	gentamicin	M13771
		ant(6)-Ia	100.0	35526..36389	streptomycin	KF421157
		lsaE	100.0	39968..41452	lincomycin	JX560992
		lnuB	100.0	41506..42309	lincomycin	JQ861959
		ant(6)-Ia	99.9	4913..5716	streptomycin	AF330699
		aph(3')-III	100.0	6348..7142	kanamycin	M26832
		ermB	100.0	7892..8629	erythromycin	U86375
	Plasmid (pEF36_2)	*ermB*	99.7	16543..17280	erythromycin	JN899585
		cfr(D)	99.9	23968..25041	chloramphenicol	NG_067192

		poxtA	100.0	25195..26802	chloramphenicol	MF095097
		fexA	99.6	28404..29831	chloramphenicol	AJ549214
EFS108	Chromosome	*optrA*	100	48604..50571	linezolid	KT862784
		ermA	100.0	53863..54456	erythromycin	EU348758
		ant(9)-Ia	100.0	54626..55408	spectinomycin	X02588
		lsaA	99.3	2726783..2728279	lincomycin	AY225127
	Plasmid (pEF108_1)	*tetM*	96.5	2422..4341	tetracycline	EU182585
		tetL	100.0	4535..5911	tetracycline	M29725
		cat	98.9	6953..7600	chloramphenicol	U35036
		ermB	100.0	15729..16466	erythromycin	X72021
	Plasmid (pEF108_2)	*cfrD*	99.9	17354..18427	chloramphenicol	NG_067192
		poxtA	100.0	18581..20188	chloramphenicol	MF095097
		fexA	99.7	21790..23217	chloramphenicol	AJ549214
